# Soil Microbial Communities in Lemon Orchards Affected by Citrus Mal Secco Disease

**DOI:** 10.3390/genes15070824

**Published:** 2024-06-21

**Authors:** Alexandros Mosca, Giulio Dimaria, Daniele Nicotra, Francesco Modica, Maria Elena Massimino, Antonino F. Catara, Giuseppe Scuderi, Marcella Russo, Vittoria Catara

**Affiliations:** 1 Department of Agriculture, Food and Environment, University of Catania, 95123 Catania, Italy; alexandros.mosca@gmail.com (A.M.); giulio.dimaria@unict.it (G.D.); daniele.nicotra@phd.unict.it (D.N.); francesco.modica@unimore.it (F.M.); mariaelenamassi@icloud.com (M.E.M.); 2 Agrobiotech Soc. Coop., 95121 Catania, Italy; antoninocatara@virgilio.it (A.F.C.); gscuderi@agrobiotech.it (G.S.); mrusso@agrobiotech.it (M.R.)

**Keywords:** *Citrus limon*, *Plenodomus tracheiphilus*, soil microbiome, qPCR, bacterial and fungal communities, amplicon-based metagenomics

## Abstract

Mal secco is a vascular disease of citrus caused by the mitosporic fungus *Plenodomus tracheiphilus*. Soil containing infected plant material constitutes an inoculum source for root infections. In this study, the soil bacterial and fungal communities of five lemon orchards located in Syracuse Province (Sicily, Italy) affected by mal secco were analyzed. Soil samples were collected under lemon tree canopies and subjected to total genomic DNA extraction. The fungal DNA was detected through qPCR in all orchards, with variable concentrations. Bacterial and fungal communities were profiled using 16S and ITS amplicon-based high-throughput sequencing, respectively. According to our results, the relative abundances of the most represented bacterial phyla (e.g., Proteobacteria, Actinobacteriota, Acidobacteriota) changed across the orchards, while in the fungal community, the phylum Ascomycota was dominant, with Basidiomycota and Mortierellomycota abundances fluctuating. On the whole, β diversity analysis showed significant variation in the composition of the soil microbial communities across the orchards. This result was confirmed by the analysis of the core community (taxa present at ≥ 75% of total samples), where putative beneficial bacteria resulted in significantly enriched fungus-infected soil samples, suggesting complex microbial interactions. Our findings shed light on the composition and diversity of the soil microbiome in lemon orchards with the occurrence of mal secco infections.

## 1. Introduction

Citrus represents one of the most important fruit tree crops worldwide. Lemon (*Citrus limon*) is the third most significant species in terms of economic importance, with a global production, together with limes, accounting for 21.5 million tons worldwide (FAOSTAT, 2022) [1]. Lemon is particularly susceptible to Citrus mal secco, a highly destructive tracheomycotic disease caused by the mitosporic fungus *P. tracheiphilus* (Petri) Gruyter, Aveskamp, and Verkley (syn. *Phoma tracheiphila* (Petri) Kantschaveli and Gikashvili). This disease causes significant damage to lemon production and tree heritage [2,3] and is a limiting factor for lemon cultivation (EFSA PLH, 2014). Other very susceptible species are lime (*C. aurantifolia*), citron (*C. medica*), and bergamot (*C. bergamia*). Occasionally, sweet orange (*C. sinensis*), grapefruit (*C. paradisi*), clementine mandarin (*C. clementina*), and tangerine (*C. reticulata*) are affected via rootstock infection. The worldwide prevalent lemon rootstocks—sour orange (*C. aurantium*), rough lemon (*C. jambiri*), volkamer lemon (*C. volkameriana*), and alemow (*C. macrophilla*)—are very susceptible [3].

The fungus is distributed across the countries of the Mediterranean and Black Sea basins and is of quarantine concern for various global regional plant protection organizations (EPPO Global Database, https://gd.eppo.int/taxon/DEUTTR/distribution, accessed on 22 April 2024).

*P. tracheiphilus* penetrates the tree through wounds in both the canopy and roots, and infections vary in intensity and progression depending on the host’s susceptibility (scion and rootstock), the virulence and site of pathogen penetration, and pedoclimatic factors [3,4]. The disease causes leaf and shoot chlorosis, wilting of leaves, and leaf fall. The shoots, branches, and trunk show basipetal desiccation until the death of the plant [2,3,4,5]. When the fungus penetrates through wounded roots without any external symptoms at first, it can persist for several years in the inner layers, and the disease initially progresses very slowly (“mal nero” syndrome). If the fungus penetrates the main roots, the disease progresses very quickly (“mal fulminante” syndrome) [3].

Despite extensive research, there are no effective standalone methods for controlling the disease. However, an integrated approach combining agronomic practices and foliar pesticide application offers some level of infection containment. Ongoing research is exploring biological mitigation through biocontrol agents and defense-inducing bacterial sprays on the canopy [6,7,8,9]. Many studies have been carried out on the genetic improvement of lemon cultivars, aimed at combining resistance to mal secco with the satisfaction of agronomic and qualitative traits [10,11].

No strategies have been developed, however, to control soil inoculum. Infected plant stems and leaves that have fallen to the ground contribute to increased *P. tracheiphilus* inoculum in the soil, as do the residual roots of uprooted lemon trees [12,13,14,15,16,17]. Leaving pruning material on the ground has shown that soil containing infected wood can act as a source of inoculum for wounded citrus roots for more than four months [12]. The pathogen’s ability to survive in infected host plant debris on the orchard floor can range from 30 days to one year, depending on the soil type [18]. Specific trials also indicate that the soil type does not substantially influence its infective capacity, although clay soil exhibits slightly lower infectivity [18]. Quantitative qPCR has revealed that fungal propagule density is higher in the upper soil layers (first 10 cm), decreasing in the summer and increasing in the autumn [17,19]. Several authors have shown a possible competitive role for antagonistic fungi isolated from soil [3].

Soil microbial communities are the largest reservoir of known biological diversity and can serve as a potential source of phytopathogens [20].

The inventory of fungal plant pathogens in 42 agricultural soils using amplicon-based metagenomics showed the presence of potential root- and shoot-infecting fungi and that the composition of pathogens in soil is driven by different environmental factors including pH, soil type, crop history, litter, saprotrophic fungi, and spatial patterns, the latter referring to the arrangement of disease entities relative to each other and to the architecture of the host crop [21].

Nevertheless, the soil and rhizosphere also contain beneficial microbial communities that enhance soil suppressiveness and can play a crucial role in managing soil-borne pathogens and disease control [22,23]. Like other crops, soil microbial communities participate in root microbial assembly in citrus, as specific microbes are selected from the soil and recruited into the rhizosphere, with root exudates acting as signal molecules and nutrition sources. Citrus recruits rhizosphere microbial communities from the extensive soil biodiversity, and the microbiome is considered to play a significant role in maintaining citrus health [24,25,26,27,28,29]. An increasing number of studies, primarily utilizing amplicon-based metagenomics and also whole-genome shotgun metagenomics, have delved into the microbial communities associated with citrus [25,26,30,31]. Soil microbial communities from citrus orchards have been demonstrated to be influenced by seasonality, plantation age, soil pH and content of nutrients, and agronomic practices such as soil and water conservation measures, mulching, cover cropping, intercropping, minimum soil tillage, and treatment of soil with bacterial biocontrol agents [32,33,34,35,36,37,38,39]. It has been observed that core microbial members of the citrus rhizosphere are strongly associated with citrus hosts from different geographical locations, and a host-driven recruitment of microorganisms from soil based on the selection of particular traits has been postulated. It has been shown that *P. tracheiphilus* infections through leaves or roots can interfere with the microbial communities of sour orange by inducing a depletion of potentially beneficial taxa (e.g., *Pseudomonas* and *Burkholderia*) in the rhizosphere and a rewiring of co-occurrence networks in the root endosphere [40].

In this study, we employed amplicon-based metagenomics to investigate soil microbial communities in lemon orchards affected by mal secco disease, focusing on the potential reservoirs of *P. tracheiphilus* inoculum. By evaluating the composition and diversity of bacterial and fungal communities across different orchards, our research underscores the ecological significance of understanding soil microbiomes. This knowledge is crucial for identifying potential microbial markers and developing strategies for sustainable disease management and enhanced orchard health.

## 2. Materials and Methods

### 2.1. Study Site

The experiment was conducted in five commercial lemon orchards located in Syracuse Province, where lemons with the Protected Geographical Indication ‘Siracusa’ are cultivated (Table 1). More specifically, there were four locations: Sant’Elia (37°00′00.8″ N 15°12′56.8″ E); San Michele (36°59′49.4″ N 15°14′25.8″ E); Cuba (36°58′54.9″ N 15°14′17.7″ E); Bonavia (36°59′14.9″ N 15°13′31.7″ E). The study area belongs to a Mediterranean climate characterized by mild winters and hot summers (Csa Köppen climate classification) with a mean annual precipitation of 544.4 mm and about 57% of the rainfall occurring in the autumn and winter seasons (October–January). The average annual temperature is 17.4 °C and the maximum (38.8 °C) and minimum (4.2 °C) temperatures occur in August and January, respectively. The average annual sunshine hours are 2753.4, and the average annual frost-free period is 222 days. The field soil was medium textured (50% sand, 30% silt, and 20% clay) and calcareous, with pH 7.8–8.2.

### 2.2. Soil Sampling

In each orchard, three soil samples (5 g each) were collected under the canopy projection area of lemon trees. A 5 cm layer of surface soil was removed and bulk soil samples without any detrital material (e.g., plant residues and rocks) were collected at 10–20 cm depth, where soil temperature was between 26 and 32 °C, using a steel spatula sterilized with 30% (*v*/*v*) household bleach between each sample. Samples were collected at least 30 cm from the tree crown drip waterline. In one orchard (CB_CU), soil samples were also collected around the planting hole of plants uprooted due to severe mal secco infections. Soil samples collected under the canopy or around the planting hole of the same plant were considered as biological replicates. Each soil sample was then mixed thoroughly, passed through a 2 mm sieve, and the material was collected in 50 mL sterile centrifuge tubes (Corning, Glendale, AR, USA).

The soil samples were kept in a cooler with ice, transferred to the laboratory, and immediately processed for total genomic DNA extraction.

### 2.3. DNA Extraction and Quantitative Detection of P. tracheiphilus

Total genomic DNA was extracted from 250 mg aliquots of each soil sample using a DNeasy PowerSoil Pro Kit (Qiagen, Hilden, Germany), following the manufacturer’s instructions. A NanoDrop 1000 spectrophotometer (Thermo Scientific, Wilmington, DE, USA) was used to estimate DNA concentration and quality.

The detection and quantification of *P. tracheiphilus* DNA were performed according to a species-specific qPCR protocol, using primers GR70 (5′-GTACCGTACGCCTTGGGGAC-3′) and GL1 (5′-AGAAGCGTTTGGAGGAGAGAATG-3′) and the probe PP1 (5′-FAM-CACGCAATCTTGGCGACTGTCGTT-BHQ-3′) [16]. A blank with ultrapure water replacing DNA and positive controls (DNA dilutions of *P. tracheiphilus* isolate PVCT Pt57) were included. For fungal DNA quantification, a standard curve was constructed using 10-fold serial dilutions in sterile distilled water of PVCT Pt57 DNA (100 ng μL^−1^) by plotting cycle threshold (Ct) values versus the logarithm-transformed DNA concentration values of each dilution. The presence of living *P. tracheiphilus* propagules in the soil was assessed in a subset of samples by plating soil suspensions on a semi-selective medium (PAR) containing carrot agar (CA; 300 g carrots, 1% agar, distilled water to 1 L) supplemented with pentachloronitrobenzene (1000 mg L^−1^), ampicillin (500 mg L^−1^), and rifampicin (10 mg L^−1^) [41]. Plates were incubated at 23 °C ± 2 for five days.

### 2.4. Bioinformatics and Statistical Analysis

For 16S and ITS amplicon sequencing, soil DNA aliquots from three soil samples collected under the same plant or around the same planting hole were mixed at equimolar amounts. Library preparation and amplicon sequencing were conducted at IGA Technology Services (Udine, Italy), as described in Dimaria et al. (2023) [40]. Sequencing of 16S and ITS libraries was performed on an Illumina NovaSeq 6000 platform (Illumina, San Diego, CA, USA). Primers 16S-341F/16S-805R [42], targeting V3–V4 hypervariable region of the bacterial 16S rRNA gene, and ITS1/ITS2, targeting the ITS1 region of the fungal rRNA operon, were used for bacterial and fungal community profiling, respectively [43]. Peptide nucleic acid (PNA)-clamping was applied during 16S rRNA amplification to block the amplification of 16S rRNA gene sequences from plant chloroplast and mitochondria. Merging forward and reverse reads, quality filtering and trimming and Amplicon Sequence Variants (ASVs) generation were performed using DADA2 (v. 1.26.0) [44] in R (v. 4.0.2) [45]. The taxonomic assignment of ASVs was performed using the 16S SILVA 138 [46] database for 16S reads, whereas the UNITE database was considered for ITS reads (version 9.0, all eukaryotic dynamic) [47]. Plant-related (e.g., chloroplast and mitochondria) and unassigned ASVs were filtered out from the 16S ASV table. α and β diversity analysis was performed using the phyloseq package (version 3.17) in R (v. 4.0.2), for microbial communities in the soil of orchards where mal secco disease had been recorded. In order to evaluate the diversity within each sample, α diversity was estimated considering the Chao1 richness and Shannon diversity indices. Statistical significance of α diversity was analyzed using the Kruskal–Wallis test, performing a pairwise comparison between each group of orchards. β diversity was assessed through the Bray–Curtis dissimilarity values and depicted with a Principal Coordinate Analysis (PCoA) to evaluate diversity within each group and among the groups of samples. The PERMANOVA test was conducted to assess statistical significance between each group of samples through vegan [48] in R. Differential abundance analysis was performed with DESeq2 (v. 1.40.2) [49] to detect the enriched and depleted bacterial and fungal genera in two groups of samples: the first group was represented by CA_SM, CA_SE and CB_CU, and the second represented by MAZ_SE and MAZ_BO. Core microbiome analysis was conducted through the microbiome R package (v. 4.3.1) [50], in order to find bacterial and fungal genera with a prevalence of at least 75% across the samples. Co-occurrence network analysis was performed considering the Spearman correlations among bacterial and fungal genera with the *Plenodomus* genus. A bi-partite network was generated through Cytoscape (v. 3.9.1) [51].

### 2.5. Analysis of Plenodomus sp. Sequences

The *Plenodomus* sp. ASV obtained from this study was aligned using MUSCLE in MEGA 11 [49] with reference sequences of *P. tracheiphilus* strains and *Plenodomus* spp. retrieved from the UNITE v. 9.0 database [47] and of representative species within the same order, Pleosporales. *Phaeosclera dematioides* CBS:157.81 was used as outgroup. A cladogram was generated by using the Neighbor-Joining method [52] and the evolutionary distances were computed using the Jukes–Cantor method [53]. The analysis was performed with 2000 bootstrap replications.

## 3. Results

### 3.1. Quantitative Detection of P. tracheiphilus in Soil by qPCR

qPCR with species-specific primers determined the amplification of *P. tracheiphilus* DNA from field soil samples, with cycle threshold (Ct) values ranging from 24.0 to 37.7 (Table 2).

A standard curve for the absolute quantification of *P. tracheiphilus* DNA was constructed. The curve was linear over seven logarithmic units of fungal DNA concentrations ranging from 100 (Ct 15.8) to 0.0001 (Ct 35.5) ng µL^−1^, with a correlation coefficient (R2) of 0.992. Although some samples with a lower DNA concentration (0.00005) were positive, with a Ct of 36–37, we only considered positive samples within the range of quantification, the lowest threshold value being 0.1 pg of DNA µL^−1^ (Ct 35.5) (Table 2). Therefore, positive samples (79/105) had a Ct value of between 24.0 and 35.5, while 26/105 samples were negative (Ct > 35.5) (Table 2). Based on the interpolation of Ct values with the standard curve, the amount of *P. tracheiphilus (Pt)* DNA per gram of soil was calculated for each sample (Table 2). All CB_CU samples collected under citrus tree canopies and MAZ_BO samples were positive, with variable DNA concentrations, while the lowest number of positive samples (3/18) and the lowest DNA concentrations (243–330 pg DNA/g soil) were found in MAZ_SE (Table 2). The fungus was also isolated on semi-selective medium from at least one sample per orchard (Table 2).

*Plenodomus* reads were detected by ITS amplicon sequencing in all samples, with the exception of MAZ_SE (Table 2).

### 3.2. α and β Diversity

Illumina sequencing of the bacterial 16S rRNA gene and the fungal ITS region produced 14,726,828 and 27,004,022 sequences, respectively. After discarding chimeras, singletons, chloroplasts, and non-microbial reads, 5,988,828 bacterial and 4,520,641 eukaryotic reads remained. Fungal reads represented 70.6% of these, representing 3,191,287 reads.

α diversity, represented by Chao1 (richness) and Shannon (diversity) metrics, was analyzed in bacterial and fungal communities. Overall, no significant differences were observed in bacterial communities’ richness across the five orchards (Supplementary Figure S1A,B). Although not statistically significant, fungal communities from the CB_CU, MAZ_BO, and MAZ_SE samples showed lower richness than the other orchard samples. Fungal communities from the MAZ_BO and MAZ_SE samples also had lower Shannon diversity values compared to the other samples (Supplementary Figure S2A,B).

**Figure 1:**
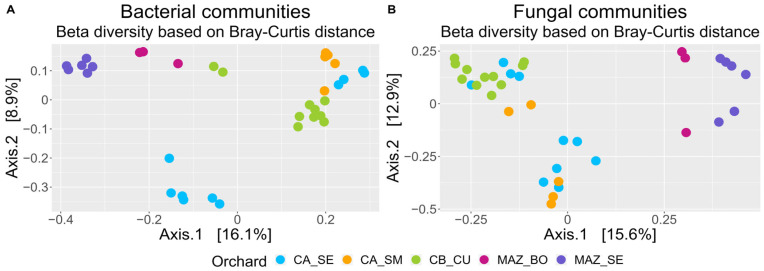
PCoA plots depicting the β diversity of the bacterial (**A**) and fungal (**B**) communities. Each color represents a specific orchard.

Principal Coordinate Analysis (PCoA), performed on the bacterial and fungal communities and based on Bray–Curtis dissimilarity distances to assess the β diversity, showed a significant division (*p*-value = 0.01) in microbial communities among the MAZ_SE and MAZ_BO orchards compared to the rest of the samples (Figure 1A,B).

Within the MAZ_SE and MAZ_BO groups, except for one sample in the MAZ_BO group, none of the samples contained reads belonging to the *Plenodomus* ASV (Table 2). Moreover, regarding the bacterial communities, a distinct separation of two groups was observed in the CA_SE orchard. In particular, the CA_SE samples in which the highest number of *P. tracheiphilus* reads were observed formed a distinct cluster.

### 3.3. Analysis of Soil Microbial Communities

**Figure 2:**
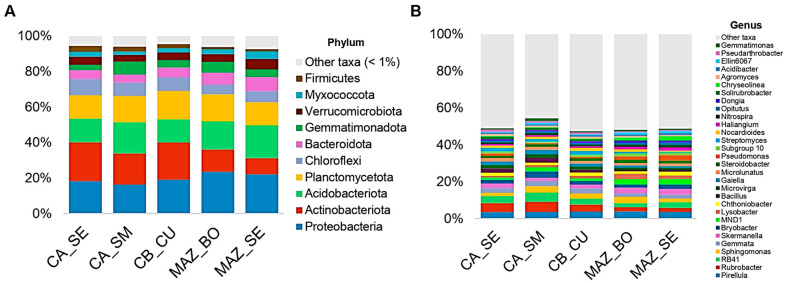
Relative abundance of the bacterial phyla (**A**) and genera (**B**).

Bacterial communities contained representatives of 39 phyla. Proteobacteria, Planctomycetota, Actinobacteriota, and Acidobacteriota constituted more than 60% of the total composition change between soil samples (Figure 2A). Planctomycetota was the most abundant phylum in CB_CU, accounting for 20.1% of the community, and Proteobacteria was the most abundant phylum in both MAZ_SE and MAZ_BO, with a relative abundance of 22% in each. The relative abundance of the 30 most abundant bacterial genera, accounting on average for 50.5%, is represented in Figure 2B. *Skermanella*, *Lysobacter*, *Pseudomonas*, and *Dongia* were more abundant in MAZ_BO (3.1%, 2.9%, 1.8%, and 1%, respectively) and MAZ_SE (3%, 1.9%, 2.9%, and 1%, respectively) than in the other three orchards (Figure 2B). In the CA_SE, CA_SM, and CB_CU samples, *Rubrobacter* (4.8%, 5.4%, and 3.8%, respectively), *Streptomyces* (1.9%, 1.4%, and 1.1%, respectively) and *Solirubrobacter* (1.4%, 1.3%, and 1.1%, respectively) were more abundant and belonged to the Actinobacteriota phylum. *Chryseolinea*, the only genus of the Bacteriodota phylum present in the top 30 genera, was more abundant in MAZ_BO (1.29%) and MAZ_SE (2.1%), whereas *Bacillus* was the only Firmicutes more abundant in CA_SE (2.2%), CA_SM (2.6%), and CB_CU (1.5%).

The taxonomic identification of the ASVs obtained through the metagenomic analysis of ITS sequences with DNA barcoding primers also revealed the composition of various kingdoms present in the soil samples. The eukaryotic kingdoms identified in the sequence pool were ranked in order of relative abundance as follows: Fungi (85.6%), Viridiplantae (5.4%), Stramenopila (3.4%), Alveolata (2.2%), Metazoa (1.8%), and Rhizaria (1.6%), together comprising 99.8% of the total (Supplementary Figure S3). In Stramenopila, the Oomycota phylum was dominant, showing a relative abundance > 80% in the CA_SE and CA_SM orchards and >60% in CB_CU, MAZ_SE, and MAZ_BO (Supplementary Figure S4).

**Figure 3:**
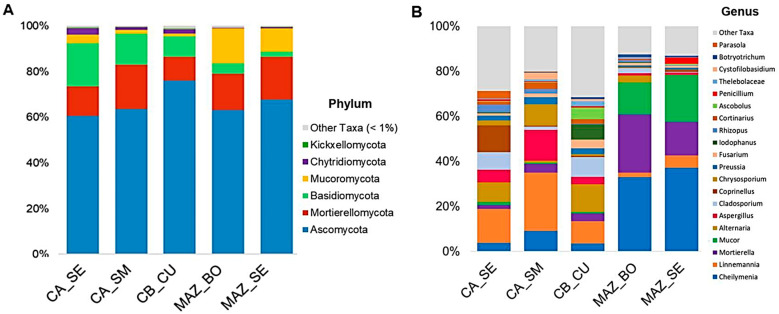
Relative abundance of the fungal phyla (**A**) and genera (**B**).

Concerning the fungal communities, 18 phyla in total were present across the samples. Among them, the six most abundant phyla (>1%) were Ascomycota, Mortierellomycota, Basidiomycota, Mucoromycota, and Kickxellomycota (Figure 3A).

The relative abundance of Ascomycota was >60% across all of the samples, whereas Basidiomycota showed the highest relative abundances in the CA_SE (18.8%) and CA_SM (13.6%) samples and the lowest in the MAZ_SE (2.3%) and MAZ_BO (4.7%) samples. On the other hand, Mucoromycota showed a significant variation between orchards in “MAZ” (where the relative abundance was at 10% in MAZ_SE and 15.2% in MAZ_BO) and in “CA” (3.88% in CA_SE and 1.22% in CA_SM) (Figure 3A). The fungal communities from the MAZ_BO and MAZ_SE samples were different from those of the CA_SE, CA_SM and CB_CU samples. In particular, in the samples from MAZ_BO and MAZ_SE, *Cheilymenia* (Ascomycota) was the most abundant genus (33% and 37.1%, respectively), along with *Mortierella* (Mortierellomycota, 25.9% and 15%, respectively) and *Mucor* (Mucoromycota, 14.1% and 21%, respectively) (Figure 3B). *Linnemania* (Mortierellomycota) was the most abundant genus in CA_SE and CA_SM (15.1% and 25.9%, respectively). Within the Ascomycota phylum, *Alternaria* relative abundance was higher in the CA_SE and CB_CU samples (8.8% and 12.4%, respectively) than in CA_SM, and *Aspergillus* was the most abundant genus (13.6%). The *Coprinellus* (Basidiomycota) and *Ascobolus* (Ascomycota) genera were highly abundant in CA_SE (11.9%) and CB_CU (5.1%), respectively.

### 3.4. Retrieval of P. tracheiphilus ASV and Further Taxonomic Identification

A total of 1677 reads belonging to a single ASV identified as *Plenodomus* was obtained. In all of the orchards, *Plenodomus* reads were detected through the ITS-based metagenomic analysis, with the exception of MAZ_SE (Table 2). However, the *Plenodomus* ASV was not retrieved in some of the soil samples that were positive in qPCR (Table 2). Soil samples from the CA_SE orchard accounted for the highest number of qPCR-positive samples, amount of DNA, and *Plenodomus* reads detected (Table 2). According to the BLASTN alignment, the sequence representing this ASV showed 100% identity and 100% query coverage with several *Plenodomus* strains, including *P. tracheiphilus* strains IS3_15 (GenBank accession no: MK461058.1), IS1_15 (MK461023.1), ISR3_6 (MK461002.1), ISR3_4 (MK461001.1), IS3_2 (MK460999.1), and ISR2_6 (MK460991.1)). A cladogram including the *Plenodomus* ASV identified in this study and other representative Pleosporales showed that this ASV clustered within a phylogroup comprising both *P. tracheiphilus* and *P. chrysanthemi* strains (Supplementary Figure S5).

### 3.5. Core Microbiome Analysis

**Figure 4:**
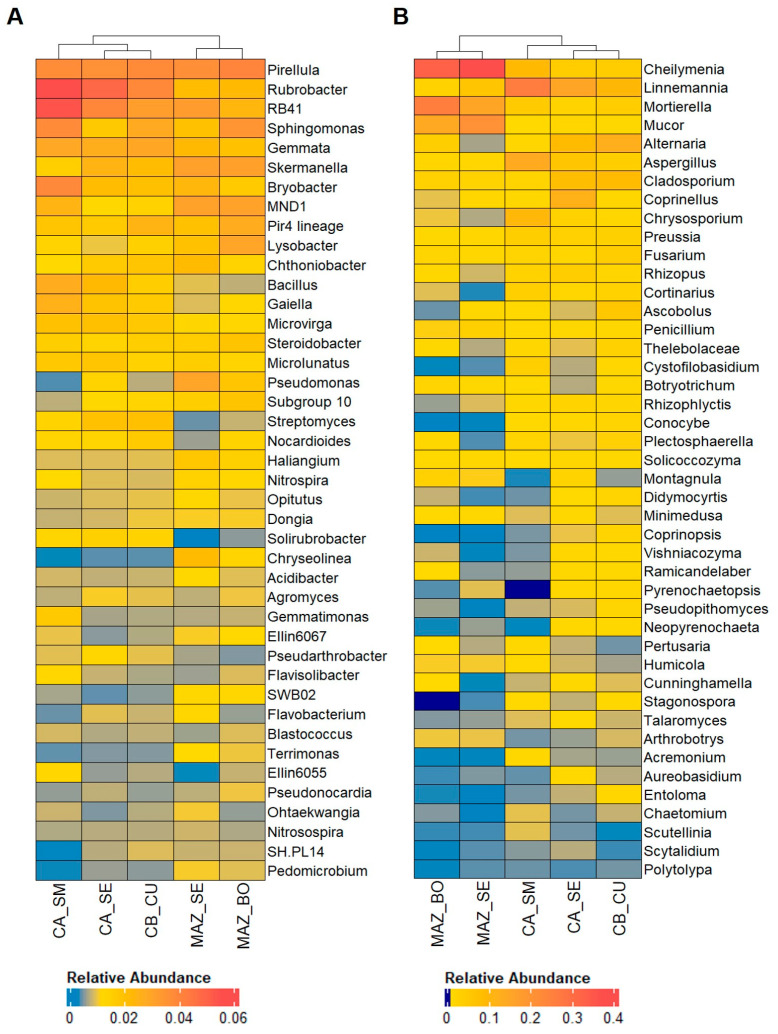
Heatmap based on the relative abundance of the core bacterial (**A**) and fungal genera (**B**) detected across the orchards. Colors from dark blue to dark red represent the lowest and highest relative abundances, respectively.

The core genera of the bacterial and fungal communities were detected with a prevalence ≥ 75% across the samples in the five orchards. According to the Euclidean distance depicted in Figure 4A,B, MAZ_SE and MAZ_BO constituted a distinct cluster in both bacterial and fungal communities.

*Pirellula*, *Rubrobacter*, RB41, *Sphingomonas*, *Gemmata*, and *Skermanella* were the most abundant core genera of the bacterial communities. The relative abundance of *Bacillus*, *Streptomyces*, and *Pseudoarthrobacter* was higher in the CA_SE, CA_SM, and CB_CU samples than in the MAZ_BO and MAZ_SE samples, which, at the same time, were characterized by the highest abundance of *Pseudomonas* (Figure 4A).

The core fungal genera belonged to *Aspergillus*, *Cladosporium*, *Preussia*, *Fusarium*, *Penicillium*, and *Scytalidium* (Figure 4B). These genera exhibited similar relative abundances across the different orchards. *Conocybe*, *Chrysosporium*, and *Rhizophlyctis* showed higher abundance in the CA_SE and CA_SM samples, while *Cheilymenia* and *Mortierella* showed the highest relative abundances in the MAZ_BO and MAZ_SE samples.

### 3.6. Differential Abundance Analysis of Bacterial Communities

The β diversity and core microbiome analyses of bacterial and fungal communities revealed a clear division of the samples into two distinct groups. As a result, a differential abundance analysis was performed to compare the CA_SE, CA_SM, and CB_CU orchards (first group) with the MAZ_BO and MAZ_SE orchards (second group).

**Figure 5:**
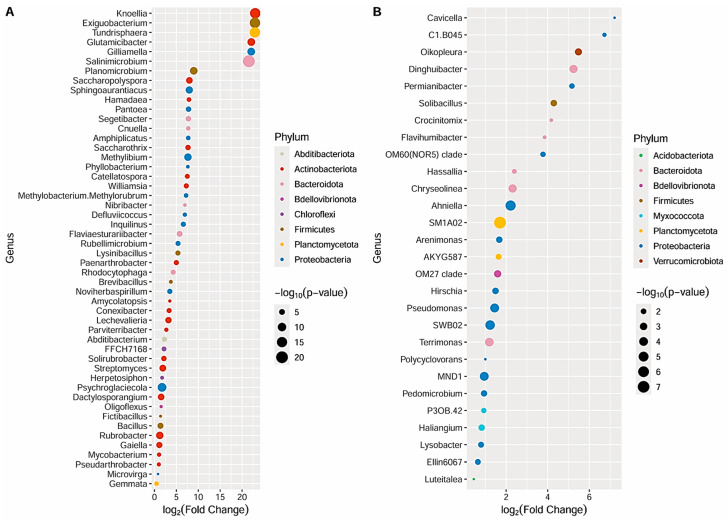
Scatter plot depicting the most significantly (*p*-value < 0.05, FDR) enriched bacterial genera in the CA_SE, CA_SM, and CB_CU orchards (the 50 most abundant) (**A**) and in the MAZ_BO and MAZ_SE orchards (**B**). The color and size of the dots depicting the log_2_(Fold Change) for each genus are based on the phylum and the negative log p-values (FDR), respectively.

As for the bacterial communities, 94 genera belonging to 13 phyla (Supplementary Table S1, Figure 5A) and 28 genera in 8 phyla were significantly enriched (*p*-value < 0.05, FDR) in the first and second groups of soil samples, respectively (Supplementary Table S2, Figure 5B).

Overall, the bacterial communities of the first group of samples were mostly represented by genera belonging to Actinobacteriota including *Glutamicibacter*, *Paenarthrobacter*, and genera detected as core members, such as *Rubrobacter*, *Streptomyces*, *Gaiella*, *Solirubrobacter*, and *Pseudoarthrobacter*, whose relative abundances were >1.10% (Figure 5A). Actinobacteriota was enriched only in this group of samples. Proteobacteria, including the genera *Gilliamella*, *Sphingoaurantiacus*, *Pantoea*, *Methylibium*, *Methylobacterium–Methylorubrum*, and *Microvirga* (the latter included in the core members), was the second most representative phylum (Figure 5A). Although with a lower number of genera, both Firmicutes (*Exiguobacterium*, *Planomicrobium*, *Bacillus*, *Lysinibacillus*, *Brevibacillus*, and *Fictibacillus*) and Bacteroidota (*Salinimicrobium*, *Flaviaesturariibacter*, *Segetibacter*, *Rhodocytophaga*, *Cnuella*, and *Nibribacter*) included six enriched genera (Figure 5A). *Bacillus* was a core member, characterized by an average relative abundance of 2.21%. Planctomycetota was represented only by *Tundrisphaera* and the core member *Gemmata* (2.64% on average). Along with Actinobacteriota, Chloroflexi and Abditibacteriota were also present only in this group of samples, with two and one genera, respectively (Figure 5A). The second group of samples showed Proteobacteria as the most representative phylum, which included the core members *Pseudomonas*, MND1, *Lysobacter*, Ellin6067, and SWB02 (Figure 4A and Figure 5B), along with *Chryseolinea* and *Terrimonas* core genera in Bacterioidota. Firmicutes was only represented by *Solibacillus*. Three phyla were present only in this group: Acidobacteria (including *Luteitalea)*, Myxococcota (P3OB-42 and the core genus *Haliangium*), and Verrucomicrobiota (*Oikopleura*).

### 3.7. Differential Abundance Analysis of Fungal Communities

**Figure 6:**
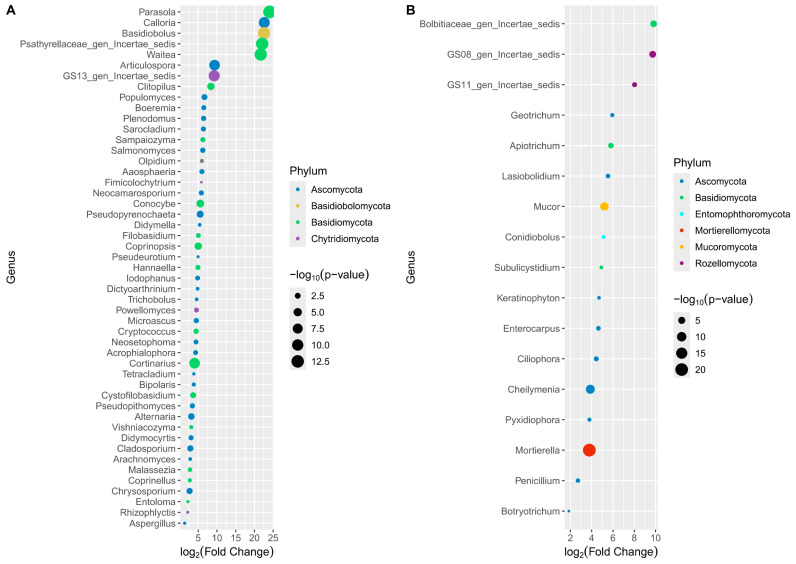
Scatter plot depicting the most significantly (*p*-value < 0.05, FDR) enriched fungal genera in the CA_SE, CA_SM, and CB_CU orchards (**A**) and in the MAZ_BO and MAZ_SE orchards (**B**). The color and size of the dots depicting the log_2_(Fold Change) for each genus are based on the phylum and the negative log p-values (FDR), respectively.

The fungal communities showed 49 and 17 enriched genera, representing five and six phyla in the first and second groups of samples, respectively (*p*-value < 0.05, FDR) (Supplementary Tables S3 and S4, Figure 6A,B). The first group of samples contained *Parasola*, Psathyrellaceae family, and *Waitea*, in the Olpidiomycota phylum, as the most significantly enriched genera, along with *Basidiobolus* (Basidiomolomycota) (Figure 6A).

Moreover, this first group of samples was characterized by 27 genera in Ascomycota, such as *Alternaria*, *Aspergillus*, *Cladosporium*, *Chrysosporium*, *Didymocyrtis*, and *Pseudopithomyces* also present as core members (Figure 4B and Figure 6A). The 16 genera of Basidiomycota were represented by core members as well, such as *Coprinellus*, *Cortinarius*, *Cystofilobasidium*, *Conocybe*, *Coprinopsis*, *Vishniacozyma*, and *Entoloma* (Figure 6A). Along with the Olpidiomycota and Basidiomolomycota phyla, Chytridiomycota was present only in this group of samples and included *Rhizophlyctis*, detected as a core genus with a relative abundance < 1%. In the second group of samples, the most enriched taxa were not identified at the genus level and were included in the Rozellomycota (present only in this group) and Basidiomycota phyla. Ascomycota, the most representative phylum, contained *Botryotrichum*, *Penicillium*, *Cheilymenia* (core members), *Pyxidiophora*, and *Ciliophora* genera, among others. *Mucor*, *Mortierella* (both core members), and *Conodiobolus* were enriched in Mucoromycota, while the Mortierellomycota and Entomophthoromycota phyla were present only in this group of samples.

### 3.8. Correlation of Plenodomus with Bacterial and Fungal Genera

**Figure 7:**
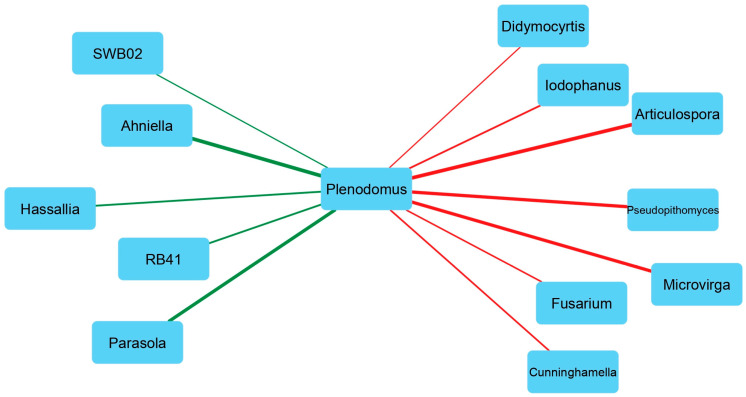
Network based on the strongest Spearman correlations of values among the *Plenodomus* with bacterial and fungal genera. Positive correlations are depicted as green edges, negative correlations as red edges. The thickness of the edges changes according to the Spearman correlation values.

In order to explore the possible interactions between the pathogen and the most abundant bacterial or fungal members in the samples where *P. tracheiphilus* reads were detected, a co-occurrence network analysis was performed. This analysis revealed statistically significant correlations (*r* ≥ 0.5 and *r* ≤ −0.5 for positive and negative interactions, respectively) between *P. tracheiphilus* and microbial genera (Figure 7).

The strongest positive correlations were revealed between *Plenodomus* and the bacterial genus *Ahniella* (Proteobacteria) and the fungal genus *Parasola* (Basidiomycota). On the other hand, the strongest negative correlations with the fungus were observed with the bacterial genus *Microvirga* (Proteobacteria) and the fungal genera *Articulospora* and *Pseudopita* (Ascomycota).

## 4. Discussion

In this study, we used a metagenomic approach to analyze the soil bacterial and fungal communities of five lemon orchards affected by mal secco disease. We identified *P. tracheiphilus* in the soil using both qPCR and metagenomics, confirming that the soil serves as a reservoir of inoculum. Additionally, culturing from some samples showed the presence of viable propagules.

In the five orchards, the bulk soil collected under the lemon tree canopies was mainly composed of six bacterial phyla: Proteobacteria, Actinobacteriota, Acidobacteriota, Planctomycetota, Chloroflexi, and Bacteroidota. All together, these six phyla represented nearly 80% of the total bacterial composition in all orchards. These phyla had also previously been reported as the most abundant in citrus soils [26]. At the genus level, the most abundant taxa reported in citrus orchards are *Streptomyces*, *Solirubrobacter*, *Bacillus*, and *Pseudomonas* [29,35,36,54]. The most represented fungal phyla are Ascomycota and Mortierellomycota, aligning with findings from Wu et al. [34] and consistently with a sampling depth of 10–20 cm. Other studies have reported higher relative abundances of Basidiomycota compared to Mortierellomycota in soil samples [29,32]. At the genus level, *Mortierella* and *Aspergillus* were the most abundant taxa across the soil samples. Biological soil crusts, or biocrusts, contain surface soil consortia of organisms (e.g., bacteria, algae, fungi, lichens, and mosses) and are commonly found in the top 0–1 cm of soil in arid and semi-arid ecosystems [55]. *Mortierella* has been associated with biocrusts in citrus orchards at 5–15 sampling depth [32]. *Aspergillus* has been shown to be enriched below biocrusts during citrus root flush and fruit maturation [56]. Moreover, *Mortierella* species are common soil and endophytic fungi known for their diverse beneficial traits, e.g., enhancing plant growth and plant defenses [57]. Additionally, they have the ability to induce xenobiotic degradation [58]. *Fusarium* species have also been detected at high relative abundance in the soil samples. This genus includes several soil-borne fungal pathogens, such as *Fusarium solani*, which is known to cause wood dry rot in citrus trees [59]. Other species within the *Fusarium* genus are responsible for significant yield losses by causing root rot and wilts in various economically important crops [60,61,62]. On the other hand, most strains are nonpathogenic soil saprophytes, and have a role in soil suppressiveness in Fusarium wilt disease [63]. Overall, microbial community composition and diversity did not change between CB_CU samples collected under tree canopies or around the planting holes of uprooted plants.

The β diversity analysis results showed that, despite the farms being situated in a very narrow geographical area, soil microbial communities, particularly the bacterial ones, tended to group based on the specific orchard rather than their location. This result suggests that the observed differences can be attributed to farming systems or specific cultivation practices in each orchard. The composition and structure of soil microbial communities in citrus orchards has been demonstrated to be affected by soil and water conservation measures such as intercropping or cover crop soil coverage [34] and organic farming practices [64].

Conversely, if the microbial communities of these farms were compared with those in other lemon-growing regions, they would likely form a single cluster due to the similar climatic conditions and soil types shared within this restricted geographical area.

β diversity analysis revealed significant differences in the overall microbial communities across the orchards, especially between a group defined by the CA_SE, CA_SM, and CB_CU orchards and another one that included MAZ_SE and MAZ_BO. This result was also consistent with the core microbiome analysis, where orchards of the same farm clustered together. Plant performance is heavily influenced by beneficial members of the core microbiome, which promote growth, enhance nutrient uptake, and bolster resistance to abiotic stresses [65]. Our study identified specific taxa that are consistently present across all sampled lemon orchards, indicating their essential role in maintaining soil health and plant productivity. Moreover, core microbiome members may also serve as an effective source of biocontrol agents against plant diseases [66]. However, it is important to note that some members of the core microbiome can also facilitate the invasion of plant pathogens, possibly by disrupting native microbial communities or altering plant defense mechanisms [67].

The relative abundance analysis of the bacterial core genera revealed two distinct groups of orchards. Therefore, to understand the differences between these groups, we compared the genera, considering both core and non-core members. The Actinobacteriota, with the genera *Streptomyces*, *Pseudoarthrobacter*, and *Gaiella*, were differentially enriched in the first group of orchards, where most of the samples were positive for *P. tracheiphilus* (CA_SE, CA_SM, and CB_CU). These bacterial genera are plant-growth-promoting rhizobacteria, antagonists of soil pathogens, and agents of denitrification processes [53,54,55,56]. Bacterial taxa belonging to this phylum were also detected as the most prevalent in soils suppressive to *F. oxysporum* wilt [68]. Other bacterial core genera enriched in the soil of these orchards have been described in disease-suppressive soils and included *Rubrobacter* [57], *Pirellula*, RB41, *Gemmata*, and *Skermanella* [69,70]. Moreover, *Bacillus*, identified as a core member of the citrus rhizosphere and a dominant genus in citrus soil [25,26], was also found to be enriched in our samples. This bacterial genus includes species with antifungal properties whose biological control functions involve various mechanisms related to the production of secondary metabolites (e.g., antibiotics, siderophores, hydrolytic enzymes, volatile extracellular metabolites, hydrogen cyanide) and competition for nutrients [71,72].

In the second group of samples, the enriched bacterial genera mostly belonged to Proteobacteria. Among them were *Permianibacter*, whose role in the decomposition of organic matter in the soil of crops of other citruses has been reported [73], and *Lysobacter*, known for its role in suppressing pathogenic fungi, oomycetes, and nematodes in soil [74]. Acidobacteria, represented by *Luteitalea*, and Myxococcota, including *Haliangium* and P3OB-42, were present only in this group of orchards. Species belonging to the genus *Luiteitalea* have been found in agricultural soils involved in denitrification [75].

Concerning the fungal communities, *Conocybe* and *Mortierella* have been reported to be engaged in the suppression of plant-parasitic nematode populations [63]. Moreover, the *Mortierella* and *Fusarium* (a core member genus in our samples) genera have been described as biomarkers of the below-ground part of mandarins grafted on ‘Carrizo’ rootstock [29].

The co-occurrence network analysis showed that as the abundance of *P. tracheiphilus* decreased, some bacterial and fungal genera abundance significantly increased. These negative correlations may represent microbial interactions such as antagonism or competition [76], which are characteristic of microbial biocontrol agents [77]. *Microvirga* emerged as one of the genera most negatively correlated to *Plenodomus.* Although there are no studies reporting potential functions of *Microvirga* as a biocontrol agent, the enrichment of this bacterial genus in suppressive soils has been reported by [78]. Similarly, the fungal genus *Articulospora* was dominant in soils characterized by the presence of pathogenic *Fusarium* species [79]. *Articulospora* spp. biocontrol activity has been reported against both bacterial and fungal phytopathogens [80,81].

A single *Plenodomus* ASV retrieved in this study was identified at the genus level according to the latest version of UNITE database (v. 9.0) [47] and clustered in a cladogram with *P. tracheiphilus* and *P. chrysanthemi* strains. In a previous study on *P. tracheiphilus*, similar results were obtained despite using a different database, necessitating analysis of sequences alongside taxonomically related taxa [40]. These species could not be differentiated based on the ITS sequence, but are pathogenic to different hosts [82,83]. In addition, *P. tracheiphilus* was detected in our soil samples using species-specific qPCR, suggesting that this ASV actually belongs to the species *P. tracheiphilus*.

However, it should be noted that, in the first group of orchards, where at least one of the three soil samples collected under each plant tested positive for *P. tracheiphilus* by qPCR, the *Plenodomus* ASV was only detected in approximately half of these samples. Therefore, these results suggest that the contribution of amplicon-based metagenomics to understanding the epidemiology of the fungus beyond the plant requires processing a sufficient number of samples. Processing with this approach may include contaminating or uninformative sequences, such as plant host DNA, which can dilute informative DNA sequences. As a result, detecting low-abundance fungal species becomes challenging, leading to reduced sensitivity in identifying specific pathogens compared to targeted amplicon sequencing methods [84].

## 5. Conclusions

In this study, variable concentrations of *P. tracheiphilus*, the fungal causal agent of citrus mal secco, were detected using real-time PCR in the soil of lemon groves in which symptoms of the disease had been observed. The fungus was present both under the tree canopy and in the soil after trees had been uprooted. The presence of living propagules was confirmed.

Soil bacterial and fungal communities, profiled using amplicon-based high-throughput sequencing, exhibited varying compositions and diversities across the orchards. β diversity analysis demonstrated that samples clustered primarily according to orchard location. Furthermore, the composition of bacterial and fungal communities differed significantly between two groups of orchards characterized by the highest and lowest presence of *P. tracheiphilus*, respectively. These findings emphasize the soil’s pivotal role as a reservoir for plant pathogens and imply potential interactions between *P. tracheiphilus* and indigenous microbial communities that could influence pathogen populations. Further exploration into enhancing these communities, through interventions such as exogenous biocontrol agents or targeted agricultural practices, may offer promising strategies for effectively managing citrus mal secco disease.

## Figures and Tables

**Table 1:** Origin of soil samples collected under the canopy of lemon (*C. limon*) trees or from areas where infected trees had been uprooted.

Orchard	Farm	Location ^b^	Scion	Rootstock	Farming
CA_SE	CA	Sant’Elia (SE)	‘Femminello Zagara bianca’	*C. aurantium*	Conventional
CA_SE	CA	Sant’Elia	‘Femminello Siracusano 2Kr’	*C. aurantium*	Conventional
CA_SM	CA	San Michele (SM)	‘Verna’	*C. volkameriana*	Conventional
CB_CU	CB	Cuba (CB)	‘Femminello Siracusano 2Kr’	*C. volkameriana*	Organic
CB_CU ^a^	CB	Cuba (CB)	‘Femminello Siracusano 2Kr’	*C. volkameriana*	Organic
MAZ_SE	MAZ	Sant’Elia	‘Femminello Zagara bianca’	*C. aurantium*	Conventional
MAZ_BO	MAZ	Bonavia (BO)	‘Femminello Zagara bianca’	*C. aurantium*	Conventional

^a^ Soil samples collected around the planting hole of uprooted lemon plants. ^b^ Locations are in the province of Syracuse (37°05′02″ N; 15°16′34″ E; 55 m a.s.l.; Italy) in a restricted area of 42 km^2^.

**Table 2:** Detection and absolute quantification of *P. tracheiphilus* by qPCR and detection by ITS sequencing in soil samples from lemon (*C. limon*) orchards.

Orchard	qPCR Positive/Total ^d^				Metagenomics Positive/Total ^e^		Culture Positive/Collection Site ^f^
	Ct	Soil Samples	Collection Site	pg DNA/g Soil	Collection Site	*Plenodomus* Reads	
CA_SE ^a^	24.0–35.5	19/21	7/7	243–293,777	5/7	5–1137	8/14
CA_SE ^b^	28.0–37.1	7/9	3/3	330–24,871	1/3	9	2/6
CA_SM	29.9–35.6	12/15	5/5	330–7697	3/5	6–59	6/10
CB_CU	31.2–34.6	15/15	5/5	423–3450	2/5	5–24	8/10
CB_CU ^c^	30.0–37.7	14/18	6/6	311–7236	2/6	4–24	4/12
MAZ_SE	35.0–36.6	3/18	3/6	243–330	0/6	/	1/12
MAZ_BO	31.8–35.3	9/9	3/3	275–2382	1/3	16	3/6

^a^ Femminello Zagara bianca or ^b^ Femminello Siracusano 2Kr grafted onto *C. aurantium*. ^c^ Soil samples collected around the planting hole of uprooted plants. ^d^ Soil samples positive with a cycle threshold (Ct) ≤ 35.5. Three soil samples were collected for each collection site (the area below citrus canopy or planting hole of uprooted plants). Range values are reported. ^e^ Soil samples in which the *P. tracheiphilus* ASV was detected. Soil DNA from the three samples of the collection sites was combined for metagenomics amplicon sequencing. Range values are reported. ^f^ Presence of *P. tracheiphilus* colonies on semi-selective medium.

## Data Availability

The raw data supporting the conclusions of this article will be made available by the authors on request.

## Supplementary Materials

The following supporting information can be downloaded at: https://www.mdpi.com/article/10.3390/genes15070824/s1, Figure S1: α diversity estimations of the bacterial communities; Figure S2: α diversity estimations of the fungal communities; Figure S3: Relative abundance of the eukaryotic communities at the kingdom level; Figure S4: Relative abundance of the Stramenopila communities at the phylum level; Figure S5: Cladogram of *P. tracheiphilus* isolates and the representative Pleosporales based on the partial ITS1 gene sequences; Table S1: Differentially enriched bacterial genera in CA_SE, CA_SM, and CB_CU orchards; Table S2: Differentially enriched bacterial genera in MAZ_BO and MAZ_SE orchards; Table S3: Differentially enriched fungal genera in CA_SE, CA_SM, and CB_CU orchards; Table S4: Differentially enriched fungal genera in MAZ_BO and MAZ_SE.
