# Supplementary material for: Soil Microbial Communities in Lemon Orchards Affected by Citrus Mal Secco Disease
Source: Genes (Basel). 2024 Jun 21;15(7):824. doi: 10.3390/genes15070824 (PMC11276235; doi:10.3390/genes15070824)
Supplement: Supplementary file 1 [file genes-15-00824-s001.zip › Supplementary Figures.pdf]

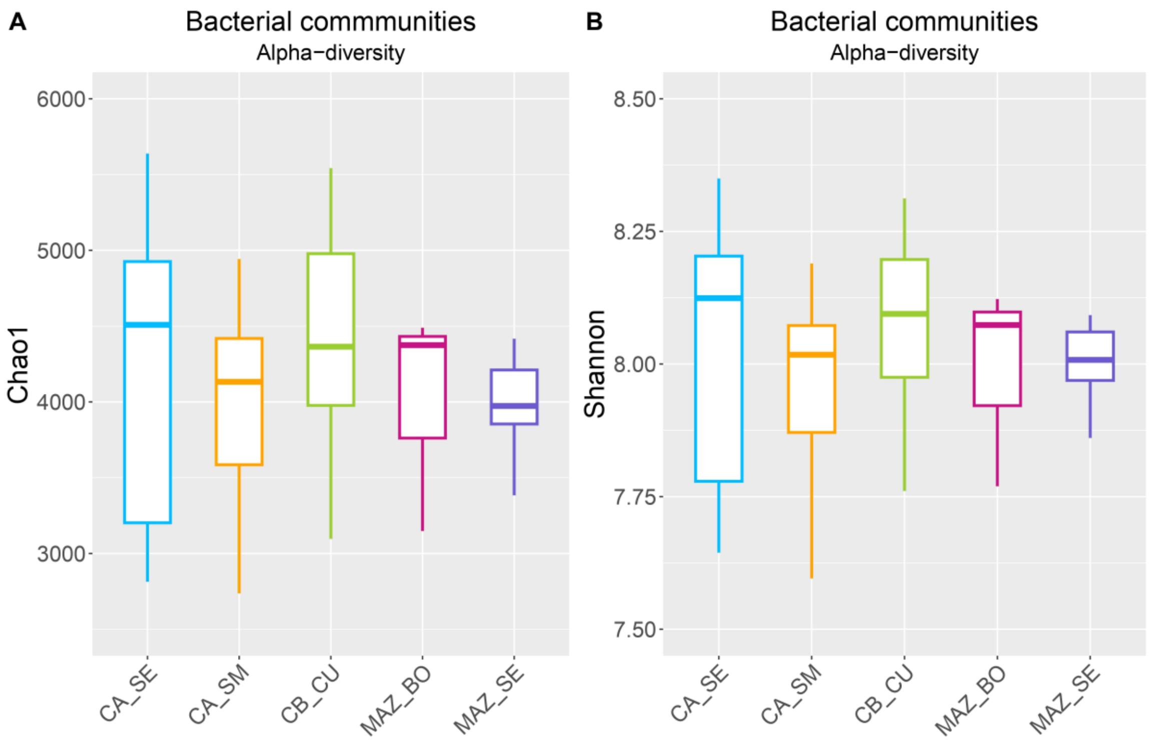

Figure S1. Alpha-diversity estimations of the bacterial communities using Chao1 richness (A) and Shannon diversity (B) indeces, respectively.

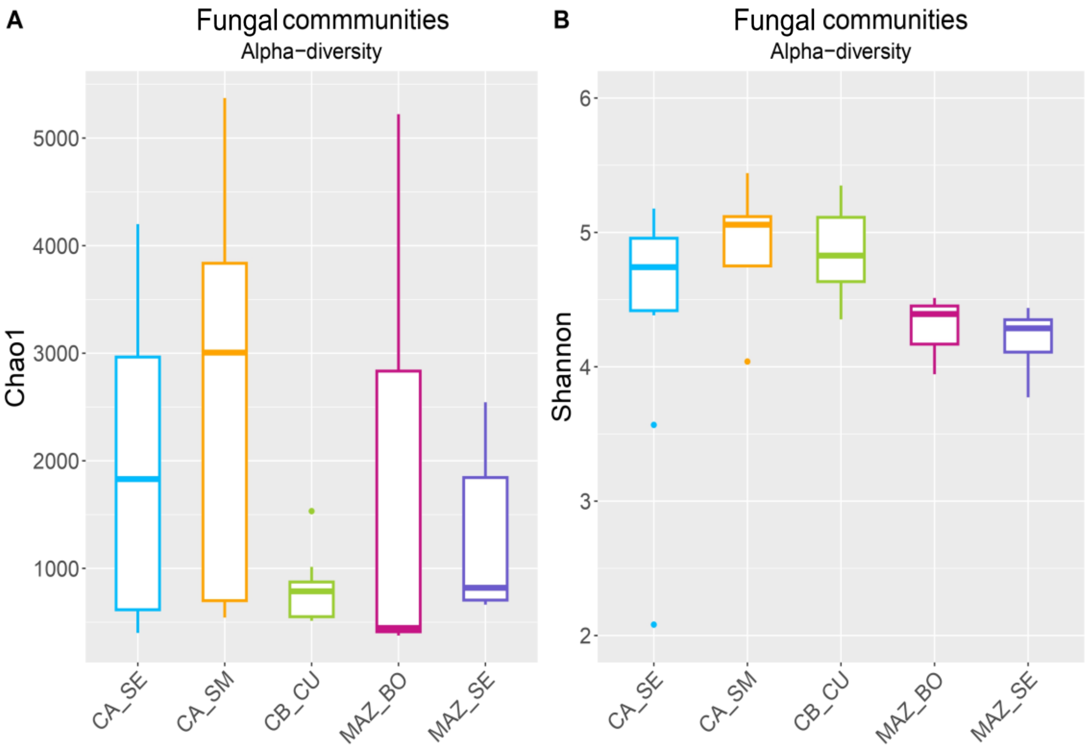

Figure S2. Alpha-diversity estimations of the bacterial communities using Chao1 richness (A) and Shannon diversity (B) indeces, respectively.

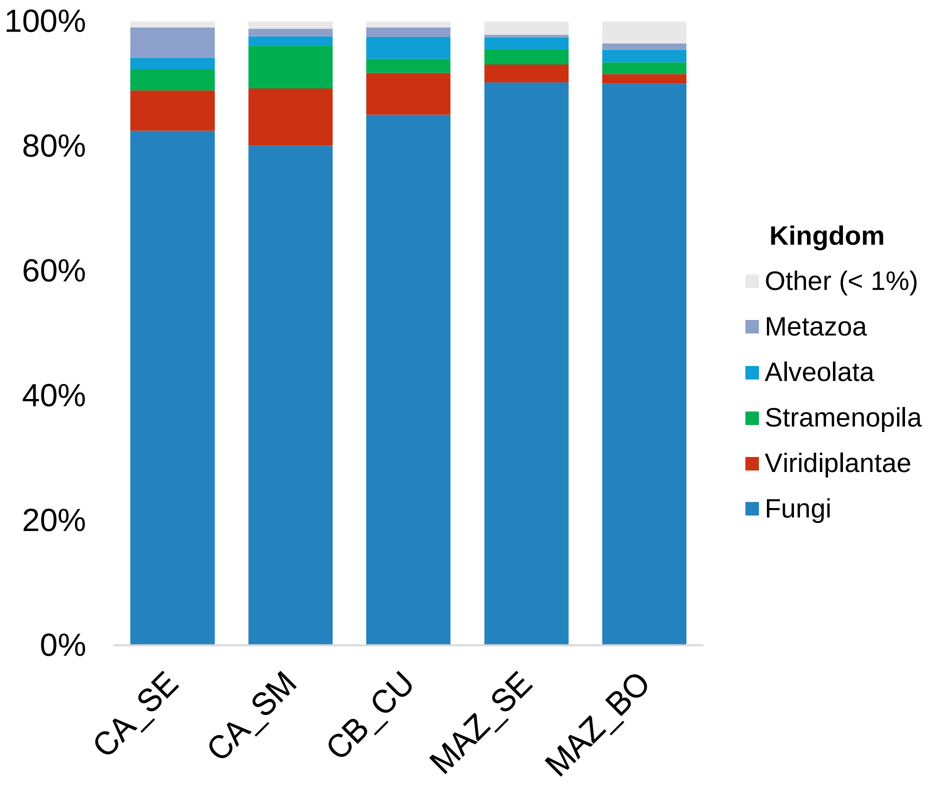

Figure S3. Relative abundance of the eukaryotic communities at the kingdom level.

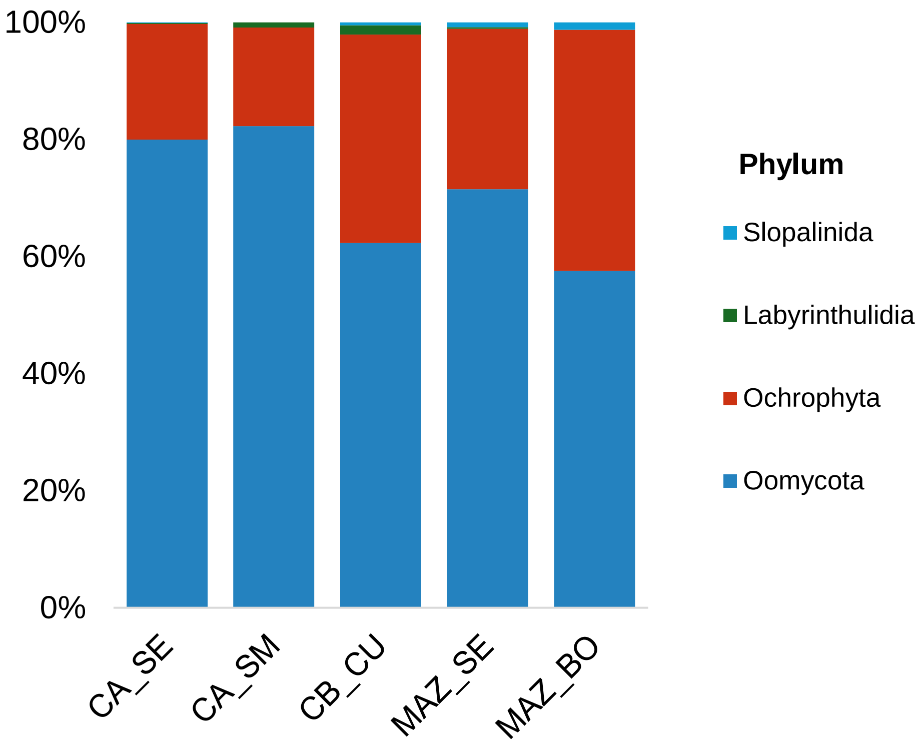

Figure S4. Relative abundance of the Stramenopila communities at the phylum level.

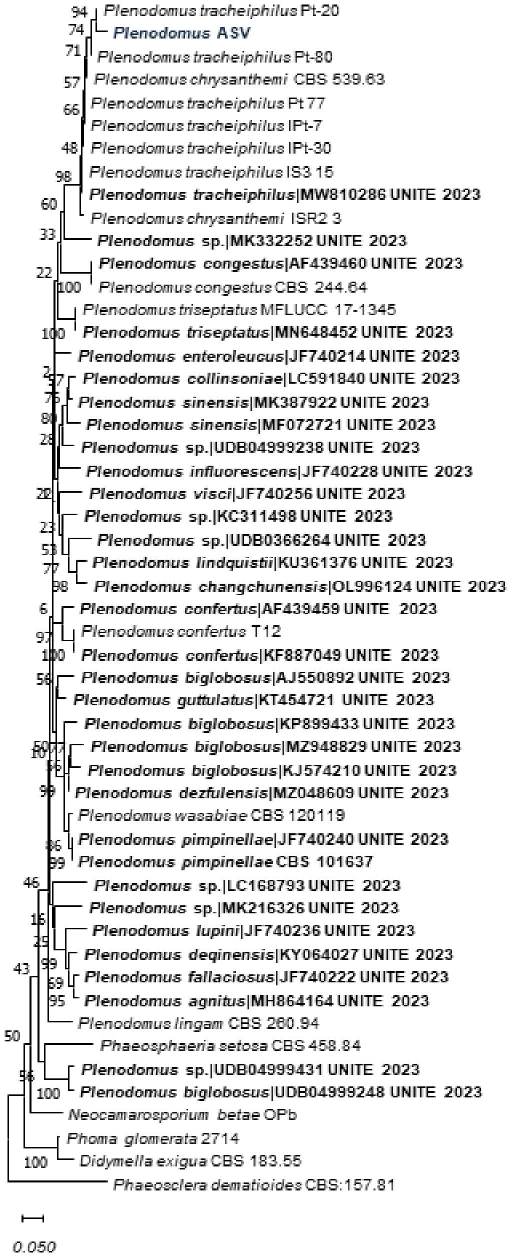

Figure S5. Cladogram of *Plenodomus tracheiphilus* isolates and the representative Pleosporales based on the partial ITS1 gene sequences. The single *Plenodomus* ASV of this study is indicated in blue and bold. *Plenodomus* spp. representative sequences retrieved from the UNITE database (v.9.0) are reported in bold. *Phaeosclera dematioides* CBS:157.81 was used as outgroup. The evolutionary history was inferred using the Neighbor-Joining method (Saitou and Nei, 1987). The tree is drawn to scale, with branch lengths in the same units as those of the evolutionary distances used to infer the phylogenetic tree. The evolutionary distances were computed using the Jukes-Cantor method (Jukes and Cantor, 1969). Evolutionary analyses were conducted in MEGA11 (Tamura et al., 2021).
